# Understanding Social Dimensions in Wildlife Conservation: Multiple Stakeholder Views

**DOI:** 10.3390/ani12070811

**Published:** 2022-03-23

**Authors:** Marcela Pimid, Mohammad Rusdi Mohd Nasir, Kumara Thevan Krishnan, Geoffrey K. Chambers, A Ghafar Ahmad, Jimli Perijin

**Affiliations:** 1 Faculty of Architecture and Ekistics, Universiti Malaysia Kelantan, Bachok 16300, Malaysia; marcela.fun@gmail.com; 2 Faculty of Agro Based Industry, Universiti Malaysia Kelantan, Jeli 17600, Malaysia; 3 School of Biological Sciences, Victoria University of Wellington, P.O. Box 600, Wellington 6140, New Zealand; geoff.chambers@vuw.ac.nz; 4 School of Housing, Building, and Planning, Universiti Sains Malaysia, Minden 11800, Malaysia; aghafar7788@yahoo.com; 5 Sabah Wildlife Department, Kota Kinabalu 88100, Malaysia; jimliowon@gmail.com

**Keywords:** wildlife conservation, social science, stakeholder conflict, social value, conservation planning framework, communication science, human–wildlife conflict, Kinabatangan, Malaysia

## Abstract

**Simple Summary:**

Garnering support from multiple stakeholders to increase the number or size of protected areas remains a key challenge for wildlife conservation efforts in Malaysia. Human–wildlife conflict often arises when local socio-economic development compromises wildlife survival due to negative landscape changes. It is essential to assess both human–wildlife conflict and human–human conflicts about wildlife, in order to promote mutually beneficial human–wildlife coexistence. This paper examines pertinent factors influencing wildlife conservation by integrating ecological and social approaches using a conservation planning framework. The findings demonstrate the importance of appraising social values to address issues such as size limits for protected areas and compensation for wildlife damage to property. It shows that monetary incentives are not the sole determinant in gaining the support of indigenous people in reporting wildlife crimes and their active participation in conservation programs. Therefore, developing effective communication with stakeholders, empowerment of rural communities, and proper appraisal of social values are all urgently needed to promote effective rural wildlife conservation programs.

**Abstract:**

Numerous studies show the importance of social understanding in addressing multifaceted conservation issues. Building on a conservation planning framework, this study examines the social dimensions of wildlife conservation in Kinabatangan, Sabah, Malaysia. It employs a qualitative approach by conducting in-depth, semi-structured interviews with sixty informants drawn from local community members, government officials, tourism operators, non-government organizations, and the private sector. Our results show that the incidence of human–wildlife conflicts has reduced in the region, but that conflicts among stakeholders themselves about wildlife still remain a significant threat for attaining successful conservation outcomes. Further stakeholder perceptions of increased wildlife numbers often contrast with actual counts returned by periodical surveys conducted by conservation agencies, e.g., showing a 30% decline of orangutans and a 29% decline of gibbon abundance. This shows that evidence-based conservation messages have not been communicated well. The study has implications for enhancing social values among conservation players, promoting local community empowerment and revising conservation awareness programs.

## 1. Introduction

The Kinabatangan area is located in Eastern Sabah, Malaysian Borneo, and recognized worldwide as a vital enclave for threatened animals [1]. For this reason, in 2017, the Sabah state government abolished a proposal to build a bridge to connect Sukau village to Litang and Tomanggong on the other side of the Kinabatangan River [2,3]. Sir David Attenborough endorsed the decision as good news because he felt it retained a safe passage for critically endangered wildlife and benefited visitors who come to see the astounding biodiversity [3]. However, indigenous communities viewed this decision as opposing their right to enhanced social and economic development [2]. It only exacerbated existing conflict between indigenous communities and animals, which had built up over the years [4,5]. This scenario demonstrates the long and painful battles over wildlife conservation in rural Kinabatangan. Hence, examining local perceptions of animal conservation programs is crucial for creating harmonious human–wildlife coexistence [6,7]. Human–wildlife conflict is a global phenomenon, and research shows there are no one-size-fits-all solutions for this problem [8].

Habitat loss is a primary cause of biodiversity loss, and has been shown to contribute to human–wildlife conflicts [9,10]. Ecological barriers such as fragmentation and loss of forest prevent effective conservation [9]. Human–tiger conflict in the Leuser ecosystem (Sumatra, Indonesia) required support from a conflict mitigation team to prevent injury to people and livestock [11]. Increased pressures to tackle wildlife issues negatively affect public support for conservation, such as limited funding, unequal distribution of conservation benefits, and weak governance [12]. These studies demonstrate that ecological barriers, social conflict, and a lack of conservation capacity impede conservation effectiveness. Biological and ecological science may provide a solid technical basis for conservation management but still may not offer a complete understanding of multifaceted conservation issues that involve people as local stakeholders [13]. Such knowledge is particularly needed for managing conservation conflicts [8,14]. Indeed, a diverse multi-discipline approach is needed because no single perspective suffices to understand the relationship between humans and nature, or to solve conflicts between biodiversity conservation and community livelihoods [13,15,16].

Conservation social science is a recent discipline and uses social science to improve conservation practice, from individual to community, and at local-to-international levels [15,17,18]. This concept combines sociology, psychology, and communication, to provide a human dimension for natural resource management [17]. Conservation social science is beneficial in examining three fundamental aspects of conservation: individual attributes (e.g., values, perceptions, and knowledge), social phenomena (e.g., socio-economics, governance, and policy), and social processes (e.g., local development and decision making); see Bennett et al. [19]. Understanding how social values guide what people perceive and how they process information can serve as a potent source of information for conflict management [20]. Human behaviors change if their values change [20,21]. Social values can influence stakeholder perceptions and their support for conservation measures [15,22,23]. Public participation in conservation programs is influenced by their win–lose perspective on wildlife management [24].

The Kinabatangan is a major global hotspot for biodiversity [25,26]. It is well-known for its abundant and diverse wildlife, including spectacular species, such as Bornean elephant (*Elaphas maximus borneensis*), Bornean orangutan (*Pongo pygmaeus*), and proboscis monkey (*Nasalis larvatus*). Many of these rare and endangered animals are on the IUCN’s RED list [27,28]. More than 80% of the lower parts of the Kinabatangan floodplain land area have been converted to oil palm plantations over the past 40 years [29]. Excessive loss of native forest due to oil palm cultivation has led to irreversible conservation issues such as habitat loss, soil degradation, fragmentation of refugia, loss of valuable species, a general decline in biodiversity, and increased human–wildlife conflict [26,29,30]. There are extensive base line studies on biodiversity distribution in this region [25,26,31]. However, previous conservation studies had only a limited understanding of the values that determine stakeholder perceptions and their support for conservation measures. The goal of this study is to examine public views of the Lower Kinabatangan Wildlife Conservation Sanctuary. We explore three pertinent questions: (1) What is the true value of Kinabatangan wildlife conservation initiatives from a stakeholder perspective? (2) How do social values influence stakeholder views and support for conservation? (3) How can an organization enhance wildlife conservation based on improved knowledge of stakeholder perspectives?

## 2. Materials and Methods

### 2.1. Conceptual Framework

The study examines stakeholder perceptions about protecting threatened wildlife species in the rural Kinabatangan landscape. We also assess how social values influence stakeholder views and conservation outcomes. Social values can be divided into three types: instrumental, intrinsic, and relational [32]. Conservation studies have advocated understanding relational values in improving relationships among people or between nature and people [32,33]. Relational values emphasize how humans can contribute to nature in order to pursue a good life [34]. These values go beyond what instrumental (people’s needs) and intrinsic (nature’s inherent worth) values can do to improve human–nature relationships. Relational values promote moral and social responsibility among people in protecting animals [15,23,34].

**Figure 1:**
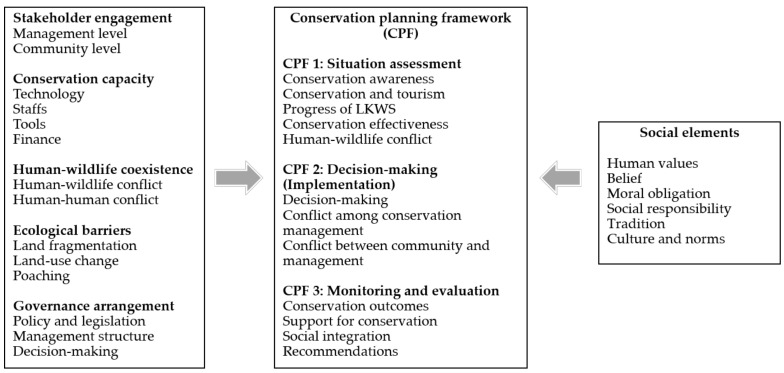
Conceptual framework of conservation planning in Kinabatangan.

This study applies a conservation planning framework (CPF) approach to examine stakeholder perceptions and conservation outcomes in the Kinabatangan (Figure 1). The basic concept of the CPF is coexistence between humans and wildlife or between groups of people [6]. Rather than focusing on the ultimate conservation goal of saving species, ecosystems, and biodiversity in a general sense, a coexistence (existing in harmony together) model improves the relationship between humans and wildlife. The CPF involves three essential stages: (1) situation assessment, (2) decision making plus implementation, and (3) monitoring and evaluation [6,35,36]. This study assesses several themes in each stage, such as conservation awareness, conflict with conservation management, and conservation outcomes. It also examines practical barriers that affect animal conservation in this region. The benefits of applying this framework include reliable and complete assessment, a decision-making process based on social evidence, and robust monitoring and evaluation leading to public communication of conservation outcomes [36]. Previous works have applied this type of CPF to integrate multiple stakeholders to better engage in coexistence with animals by focusing on human–wildlife conflict and how best to manage it [6,36]. In this study, we broaden the usage of CPF to examine how social values influence stakeholder perceptions and support for conservation, hence, adding new information to the literature on human–wildlife coexistence.

### 2.2. Study Area

**Figure 2:**
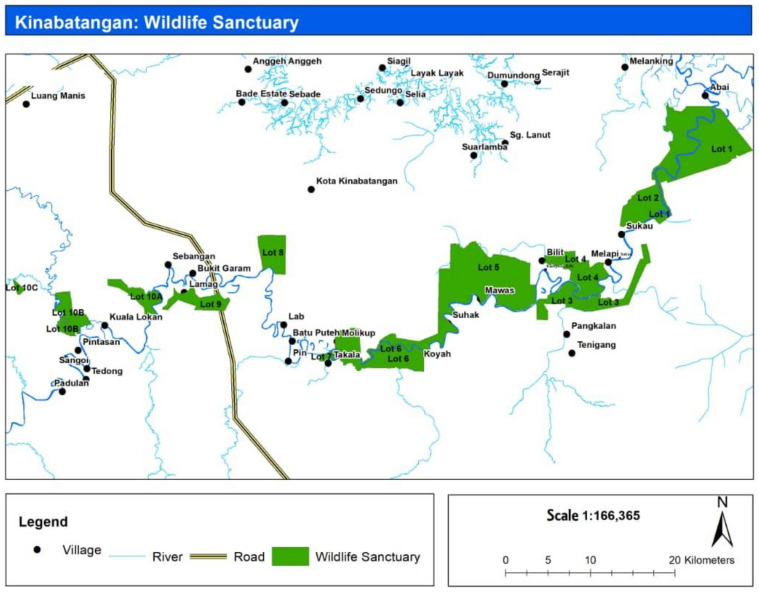
Ten lots (green) as Lower Kinabatangan Wildlife Sanctuary.

The study was conducted in the Lower Kinabatangan region. In 2005, the state government established the Lower Kinabatangan Wildlife Sanctuary (LKWS) to protect the remaining biodiversity in this area. The LKWS comprises 10 fragmented lots with a total area of 26,103.29 hectares (Figure 2). These protected forests represent various lowland habitats such as mangroves, freshwater swamps, riverine, dry-land dipterocarp, and limestone forests. It is a refuge for several globally threatened species such as the Bornean elephant, proboscis monkey, orangutan, oriental darter (*Anhinga melanogaster*), clouded leopard (*Neofelis diardi*), Storm’s stork (*Ciconia stormi*), estuarine crocodile (*Crocodilus porosus*), and eight species of hornbills (*Buceros rhinoceros*, *Rhinoplax vigil*, *Anthracoceros malayanus*, *Anthracoceros albirostris albirostris*, *Anorrhinus galeritus*, *Aceros undulates*, *Aceros comatus*, and *Rhabdotorrhinus corrugatus*) [1,37]. The forests are embedded within more than half a million hectares of agricultural land, dominated mainly by intensive oil palm plantations [38].

### 2.3. Data Collection

This study used in-depth, semi-structured interviews to obtain unique insights [39] into, and increase our understanding of, local conservation efforts [19]. We employed a pragmatic approach focused on research problems and where data collection tools were chosen to match research questions. In contrast to other paradigms (i.e., postpositivist, constructivist, and transformative), pragmatism employs techniques to understand research problems and select appropriate data collection and analysis methods without relying on any particular philosophy [40]. We conducted sixty interviews with key stakeholders in the Kinabatangan area: 38 males and 22 females comprised of 20 local community members, 14 government officials, 8 non-government organizations (NGOs), 12 local tourism operators, and 6 private oil palm plantations. At the outset of this project, we sent an official letter to the district office of Kota Kinabatangan, the Sabah Wildlife Department, the Sabah Forestry Department, and Kinabatangan village leaders to request their consent. The study employed non-probability sampling [41]. Respondent selection followed a purposive sample, using inclusion criteria that they be directly involved in conservation programs or management of conservation in Kinabatangan [42]. Participation in the interviews was voluntary. When a selected respondent rejected an interview invitation, we used a snowball sampling technique, asking the person to pinpoint an individual who had similar expertise or experience in conservation matters. Snowball sampling emphasizes the quality and depth of findings and has been applied in many conservation studies [43]. Each interview session was recorded using digital equipment and lasted between 30 and 45 min. The interviewees were assured anonymity regarding their names, positions, and affiliations. This approach allows the respondents to be open-minded and honest when answering interview questions, especially when they disagree with conservation practices or policy. Audio recordings were manually downloaded to computer file stores. They were transcribed using Microsoft Word [44]. Three individuals listened to the recordings and compared them to the Word scripts. After the transcripts were checked for accuracy, the recorded sound files were deleted [44].

To investigate whether the objectives of conservation planning were achieved in this area, we collected data on four topics. These include (1) assessment: stakeholder views on conservation programs, current conservation practices and their effectiveness in the Lower Kinabatangan; (2) decision-making: stakeholder approach to decision-making for conservation, governance, communication, conservation barriers, and community involvement in the decision-making process; (3) evaluation: monitoring of conservation programs, prosecution of conservation laws, community willingness to support and take part in conservation activities, and stakeholder willingness to collaborate in achieving conservation goals; (4) social dimensions of wildlife conservation: cultural norms, socio-economic values, beliefs, perceptions, and moral and social responsibility. These themes were selected from the CPF framework [6]. Examples of interview questions include: What do you think about the amount of wildlife in Kinabatangan? What do you think about the size of the habitat available for the animals? Are you willing to support conservation programs in the future? Details of interview questions were provided in Supplementary Material file.

As this study was exploratory, we conducted participant observation throughout the fieldwork to compare and verify the data obtained through the interviews, including writing notes and highlighting particular words that reflected the themes stated earlier [45]. We also gathered reports from government offices, the private sector, NGOs, and tourism operators, and compared these results with the findings from our interviews to triangulate our results [45,46].

### 2.4. Analysis of Qualitative Data

Data obtained from interviews, written notes, and participant observation was analyzed within a framework method as revised by Gale et al. [47], which consists of seven steps: transcription, familiarization with the interview data, coding, developing a working analytical framework, applying the analytical framework, charting data into a framework matrix, and finally, interpreting the data. We included field notes and personal observations in the framework matrix to increase the validity of data findings. The matrix was arranged systematically to compare the transcript and later for checking whether the proposed themes were supplied with sufficient evidence. This study used both deductive and inductive approaches within the framework method because we aimed to explore specific issues and offer an opportunity for respondents to give answers that were not pre-coded [48].

The analysis employed a sequential approach by initially applying a deductive technique to draw initial codes from existing literature reviews on Kinabatangan conservation and subsequently comparing them using the inductive method. This procedure generated themes based on data obtained during sampling. To identify emerging themes, three coders conducted separate coding analyses of the interview transcripts for the inductive approach. The integrated data from the inductive and deductive approaches were cross-tabulated and applied to the framework, in order to examine patterns or relationships among the categories [48]. Specific codes such as ‘crop damage’, ‘mistrust’, ‘limited finance’, and ‘lack of human resources’ were categorized into broader themes such as ‘conservation capacity’, ’conflict among stakeholders’, and ’human–wildlife conflict’. The themes derived from inductive coding were compared between the three coders to ensure consistent intercoder reliability [49]. Each coder’s coded data from Microsoft Word was exported into the Statistical Package for Social Science (IBM SPSS version 22), and intercoder reliability was measured using Krippendorff’s alpha. In cases of conflicting interpretations, some discussion between coders is necessary to identify why the interpretations conflict. These discussions inform the refinement of the coding frame to improve precision [49]. In the Kinabatangan study, a first round of independent coding was followed by a meeting where differences were discussed, the coding frame was revised, and a second round of independent coding commenced. We exercised thoughtful consideration to improve data quality, rigor, and transparency.

## 3. Results

### 3.1. Conservation Planning Framework

The study examined the conservation status of Kinabatangan with respect to three crucial aspects of the conservation planning framework, namely: (1) assessment, (2) decision making, and (3) evaluation. Regarding the situation assessment, all respondents concurred that conservation awareness had improved compared with previous years (see later in Table 1). Conservation was highly interlinked with tourism development in this area. The local support for protection was influenced mainly by employment in the conservation or tourism sectors (82%), while only a few (18%) respondents related their support to protect wildlife for the benefit of future generations. The study revealed conflicting perspectives among stakeholders on the progress of LKWS and the effectiveness of conservation programs. Whilst the majority of government officials (11 out of 14) stated that the objectives of LKWS were fulfilled, many other stakeholders (55%), such as community leaders, tourism operators, and private sectors, reported they were unsure about the progress of the sanctuary or that it had been unsuccessful (12%). The contradictory claims by the stakeholders raised a question regarding the effectiveness of the sanctuary to sustain viable populations of animals. Respondents had mixed perceptions about whether the numbers of animals had increased (62%) or decreased (38%), and 46% of them were uncertain about habitat availability.

Interviewees identified several barriers to achieving conservation goals: land fragmentation, habitat loss, deforestation, poaching, human–wildlife conflict, and conflict among stakeholders about wildlife. Human–wildlife conflict is a recurring conservation issue, caused by (1) underlying reasons such as habitat loss and fragmentation, and (2) aggravating factors related to poor design of fences, etc. (i.e., irregular maintenance and difficulties inherent in installing fences over complex and challenging landscape features). Consequently, animals must cross over human settlements, farms, and plantations to reach another patch of suitable habitat. Recent development issues include the proposed Sukau bridge project, which the state government has since cancelled. The project involves building a 350 m Sukau bridge across the Kinabatangan River, followed by a paved road to the bridge. The road connects Sukau with the coastal villagers of Litang and Tomanggong, a town that sits about 42 km to the southeast. The Sukau bridge provides the local communities with access to markets and healthcare services. However, if the bridge had been built, it might have disrupted animal migration routes within the LKWS. Based on the views of the key informants, the local communities feel that they have the right to development in their area, but also that this requires proper planning so that it brings an all-round, win–win solution, including for the wildlife.

In stage two of the CPF, we found that the decision-making behind conservation actions employed a top-down approach (70%). Discussion of conservation matters involved primarily top management and community leaders, while the local community members were only informed after the meetings. Inefficient communication and conflicts among key stakeholders were significant issues identified by them. Stakeholder conflict was categorized into two types. First, conflict at the management level includes local authorities, NGOs, and the private sector (65%). Issues that caused disputes stemmed from plantation smallholders (i.e., those having to give part of their land for a wildlife corridor), competing interests, lack of cooperation, mistrust, and a lack of understanding of personal roles. Second, a conflict between local communities and upper-level management, i.e., those people employed in local authorities, NGOs, and the private sector (78%). According to the respondents, negative attitudes in local communities, mistrust, competing interests and needs, and the fact that other stakeholders were not mutually cooperative were major factors contributing to stakeholder conflicts.

Regarding monitoring and evaluation (CPF stage three), government staff (9 out of 14) highlighted that conserving animals in the Lower Kinabatangan was negatively affected by inadequate supplies of proper technologies, tools, and finances, as well as insufficient human resources. All of these factors hamper an efficient fight against illegal encroachment. Respondents (30%) reported that lack of integrity, misconduct, and dishonesty among officials or conservation agencies was an impediment. Various organizations, such as the Sabah Wildlife Department (SWD), HUTAN-Kinabatangan Orangutan Conservation Programs (HUTAN-KOCP), and Danau Girang Field Research Center, collaborate in scientific wildlife research and monitoring. To enhance the monitoring of wildlife in LKWS, the SWD has appointed extra personnel from the HUTAN-KOCP and Danau Girang as honorary wildlife wardens (HWW), all from the local indigenous community, to enforce conservation rules. This approach gives power to selected local personnel and enables them to catch criminals that commit illegal hunting. We found the local community (62%) would change to pro-conservation if conservation practices and policies were constructed based on their perspectives, prioritized their needs, and resolved recurring conservation issues in this region. The study revealed insufficient collaboration among conservation agencies about conservation management, communication (reporting) of wildlife studies, and sharing of research outcomes. Table 2 shows major findings and examples of interview transcripts.

### 3.2. Importance of Social Integration in Solving Wildlife Conservation Issues

We found that all the contributing parties had different values, beliefs, and knowledge about wildlife and forests, which affected their perceptions and behaviors. Most people’s positive views of conservation originate from NGOs or department agencies with a mandate toward conservation. Most of these respondents feel that they have a moral obligation and a duty to protect biodiversity. Tour operators and people benefiting from the tourism industry also support wildlife conservation to sustain their economic activities. In short, we found that among those working in the tourism or conservation sectors, having a personal financial interest (via business or employment) involving wildlife or natural resources played a pivotal role in determining their support. In contrast, few locals supported conservation solely on the basis of altruistic reasons of benefit for future generations. Locals do have a firm attachment to Kinabatangan land. One identity they cultivated was stewardship virtue–their feelings that they needed to protect the land and associated resources as their duty, and preserve both components for future generations. Importantly, cultural norms (i.e., relational values) determine local support and participation, as the villagers have a tradition of hunting animals for livelihood support. Because all animal species are protected and hunting is regulated by the Wildlife Conservation Enactment 1997, Sungai traditional hunting and collecting of natural resources is currently restricted.

Major conservation initiatives valued by interviewees included the use of electrical fencing to protect villagers’ crops, wildlife corridors, conservation awareness programs, honorary wildlife wardens, and tree planting (Table 3). Government authorities do not provide compensation for wildlife damage in this region. Our results showed that the local people felt that their voluntary participation in conservation programs went unappreciated. The human–wildlife conflict could be reduced by using a non-monetary approach to appreciate the local villagers, by giving them certificates and holding acknowledgement ceremonies after participating in conservation activities. This type of initiative can motivate them to adopt non-lethal methods when animals enter their farms, and encourages them to promptly report wildlife encroachment to local authorities. Such recognition of effort increases the value and responsibility of the community, which makes them more likely to support and participate in conservation programs.

## 4. Discussion

One essential criterion in conservation is to ascertain stakeholder perceptions of scientific observations [50]. This study indicates that stakeholder perceptions are not aligned with biological observations of wildlife distribution in the Kinabatangan. For instance, stakeholders perceive that Kinabatangan animals have increased (62%), but animal surveys [25] show a reduction of orangutan (30%) and gibbon (29%) numbers. We argue that not knowing the true picture constitutes a ‘silent destruction’ and is an impediment to protecting wildlife. Ignorance suppresses any community’s capability to comprehend and respond appropriately if they receive the wrong conservation messages. However, effective conservation messages promote public support for conservation [51,52]. However, our interviews revealed that in the case of scientific wildlife results (i.e., population distribution and abundance, habitat availability, and wildlife crimes), they were not shared properly with local indigenous people during conservation awareness programs. If provided with the right conservation message, the community and private sector could become actively involved during the planning, decision making, and evaluation stages, and raise their moral beliefs and values toward pro-conservation behavior [52,53,54]. Voicing opinions for conservation practices in line with social-economic needs, and built on local knowledge, would gain traction for supporting conservation [18,55].

Human–wildlife coexistence requires a thorough understanding of both human–wildlife conflict and stakeholder conflict [6,8,54]. Human–wildlife conflict has been reduced in the LKWS, but stakeholder conflict about animals remains unresolved. Instead, it can escalate because community needs for social and economic development often cause negative landscape changes, which impact wildlife distribution [4,56]. Global measures for mitigating both conflicts across different continents show varying degrees of success [7,57]. These measures include: (1) compensation, electrical fences, and legislation for human–wildlife conflict; (2) shared understanding of differing stakeholder values regarding conservation; (3) improved transparency and trust by engaging all parties; (4) willingness of parties to recognize problems as shared and to have open discussions about them, and (5) understanding that certain actions required for solving both types of conflicts exceed stakeholder capabilities [7,57,58]. Kinabatangan authorities use electrical fences and appoint honorary wildlife wardens to address human–animal conflicts. However, major human problems, such as lacking integrity and the absence of an interactive platform to discuss conservation issues openly, lead to unsatisfactory outcomes and mistrust among stakeholders about wildlife management (i.e., cause stakeholder conflict). Contentious stakeholder conflict is all too obvious. One community member comments: “The [names withheld for reasons of confidentiality] always focus on conservation work, but never bother to listen to the villagers’ opinions and problems.” Similarly, at the conservation management level, some comments from anonymous respondents were: “Did [names withheld] mention me? and “I do not trust [several names withheld] when it involves conservation matters.” Our findings suggest that the stakeholders urgently need to reconcile their differences, establish a holistic platform for open discussion, promote understanding of human–wildlife coexistence, and recognize that no one party should be accountable for all conservation problems. They need to realize that each individual holds equal responsibility to restore positive stakeholder relationships in managing wildlife services and stakeholder needs [15,59].

Ideally, effective conservation intervention requires proper management of protected areas, technology, human resources, and a good governance structure to achieve significant outcomes [24,59,60]. However, many protected areas lack these capacities [61,62], and Kinabatangan encounters exactly such problems. A major issue in Kinabatangan is forest fragmentation and the small size of protected areas [9,25]. Therefore, the Kinabatangan stakeholders need to increase the sanctuary size and reconnect the ten lots of LKWS. These changes will enable the animals to move around, forage for foods, and breed naturally [63]. However, this approach is hard to implement because it involves multiple land-use activities, such as oil palm plantations, village-owned lands, housing compounds, and blocks of private land. Some private sectors are willing to give a small part of their land for this corridor. However, a major issue lies with people who own small amounts of private land. Some villagers only have enough land to build their houses. As encouraging as they are, our findings suggest it is far from being achieved because human factors exacerbate crimes such as poaching for food and killing animals for other resources or to protect crops. Evidence shows that even 15 years after establishing the LKWS protected area, poaching still occurs in several lots of the sanctuary [1,25].

Local perceptions of underlying reasons for wildlife decline, such as habitat loss, fragmentation, and recurring wildlife crimes (i.e., poaching, snaring, and killing), are echoed by reports and assessments carried out in the region by conservation organizations [25,31,63]. Limited conservation capacity, such as lack of advanced tools (drones), human resources, and finance in monitoring the animals and LKWS, impedes conservation effectiveness. In particular, only four SWD rangers work full-time to take care of the ten lots of LKWS. They are assisted by the HWW, who work there when there is a necessity for monitoring and enforcement [1,37]. Therefore, community cooperation is needed to immediately report wildlife crimes and intensify active collaboration from all parties to assist in wildlife monitoring, thereby reducing dependency on limited conservation capacity and mitigating wildlife crimes [64,65]. Equally important, a lack of political will and the complexity of stakeholders’ competing interests in conservation versus social-economic development have complicated conservation efforts in this region. Many non-protected areas are still being used for oil palm development, even though there is strong evidence that they are not good places to grow oil palm because of flooding [29].

We argue that conservation practices need to be revised to provide sound and achievable interventions. These can be realized through multiple disciplines and the empowerment of readily available capacity. The three stages of CPF reveal interconnection and a possibility to uplift social elements across the five components: stakeholder engagement, ecological barriers, conservation capacity, governance arrangement, and human–wildlife coexistence. Notably, Catalano et al. [59] report that human dimensions of conservation project failure, particularly in stakeholder relationships, are more commonly reported than other causes of failure, such as conservation management, communication, funding, and politics. In Kinabatangan, we found that conflict among stakeholders about wildlife and psychological experiences (e.g., trust, blame, and fear of being rejected) was a common complaint during interviews. Future conservation efforts should focus on effective stakeholder engagement and community empowerment. Indeed, achieving conservation goals depends on understanding people’s values, as much as it does on understanding biological systems [7,13,15]. In this view, the Kinabatangan stakeholders can improve their relationships by learning to reconcile competing interests and social values, discussing conservation failures, and collaborating to enhance conservation intervention.

The local indigenous community can be empowered through a socio-economic approach, by encouraging and giving equal opportunities to participate in wildlife-based tourism activities that provide income, and thus soften the impact of tight conservation rules [56,66]. A non-monetary appreciation scheme, such as providing certificates and acknowledgements to local people during and after conservation tasks, improves their motivation for participating in conservation activities. The Kinabatangan stakeholders must be able to recognize missing social values in the current conservation practice, which focuses on increasing conservation awareness, but less on advancing stakeholders’ behavioral change. Few respondents relate to animal conservation as an altruistic value, showing the pressing need to promote moral and social responsibility (i.e., relational values) among the Kinabatangan stakeholders, to improve their sense of duty to both people and animals. Indigenous tolerance for wildlife (i.e., among the unsupportive group) and human–wildlife conflict can be improved by increasing their moral values in order to develop compassion for the animals [22,67]. Including scientific information about wildlife (population trends and conservation status) in environmental awareness campaigns could improve the precarious status of most wildlife populations in the area and trigger more compassion from local communities. We urge the Kinabatangan stakeholders to advocate for the rights and recognition of the local community before, during, and after conservation programs, as a sense of recognition is crucial to garner conservation support from local communities [67,68].

## 5. Conclusions

Despite extensive conservation programs undertaken in the Kinabatangan, our findings reveal that multiple ecological and social conservation issues remain unresolved. We highlight social importance by understanding human attitudes and values as interconnected in all five components: human–wildlife coexistence, ecological barriers, governance, stakeholder engagement, and conservation capacity. Therefore, any strategy to improve the current conservation situation will be ineffective unless all of these aspects are given full attention. Positive values of stakeholders’ perceptions, interactions, beliefs, and behaviors enhance wildlife conservation outcomes, but the reverse is also true. To our knowledge, this is the first report that focuses on integrating social values from the human dimension into five essential aspects of wildlife conservation in the Kinabatangan. The future of Kinabatangan wildlife conservation lies in stakeholders’ relational values and their willingness to reconcile differences and collaborate, particularly regarding the indigenous community–as they can be contributors to, or destroyers of, sustainable conservation. The study examines perceptions from 60 key informants, but future studies should include more interviewees to acquire an in-depth understanding of the social dimensions of animal conservation.

To promote human–wildlife coexistence, understanding social factors should be taken into full consideration regarding human–wildlife conflict and human–human conflict about wildlife. In this study, the grassroots issue is the human–human conflict about wildlife, exacerbated by the lack of conservation capacity to address wildlife crimes and encroachment on the sanctuary. Considering the difficulty of increasing the Kinabatangan protected area, there is an urgent need to recognize missing human values that attune stakeholder reconciliation and collaboration and empower the community through a non-monetary approach. Stakeholders need to improve communication and transparency in sharing scientific wildlife studies to ensure that each party gets the right conservation message, encouraging them to contribute voluntarily to various conservation programs. This approach can reduce dependency on limited conservation capacity. Using the CPF as a reference, similar studies on wildlife conservation and human–wildlife coexistence can be applied elsewhere. The results could be different, but it will help to improve the understanding of conservation intervention in areas with less capacity for an ecological solution.

## Figures and Tables

**Table 1:** Results of CPF based on interviews.

Themes	Frequency (%) N = 60
CPF 1: Assessment	
1. Local conservation awareness	60 (100)
2. Conservation link with tourism	58 (97)
3. Factors influence local support for conservation	
(a) Employment in conservation or tourism sector	49 (82)
(b) Altruistic reason for benefit of future generation	11 (18)
4. Progress of LKWS	
(a) Successful	20 (33)
(b) Not successful	7 (12)
(c) Not sure	33 (55)
5. Effectiveness of conservation programs	
(a) Number of animal increase	37 (62)
(b) Number of animal decrease	23 (38)
(c) Habitat availability increase	19 (32)
(d) Habitat availability decrease	13 (22)
(e) Habitat availability (not sure)	28 (46)
(f) Human–wildlife conflict reduced	42 (70)
CPF 2: Decision-making	
1. Decision making on conservation matters (top-down)	42 (70)
2. Conflict among conservation management	39 (65)
3. Conflict between community and management	47 (78)
CPF 3: Evaluation	
1. Inadequate supply of technology, tools, finance, and human resources	32 (53)
2. Integrity in governance	18 (30)
3. Local mind-set and attitudes to obey rules	40 (67)
4. Community willingness to change for pro-conservation	37 (62)
5. Importance of social integration	
(a) Moral obligation/duties to protect wildlife	45 (75)
(b) Socio-economic importance (business/employment)	48 (80)
(c) Local culture and norms	39 (65)

**Table 2:** Results of interviews.

Themes	Codes	Examples of Interview Transcripts	Notes *
Human–wildlife conflict	Crop damage	“Elephants do not destroy our oil palm every day. But when it happens, it causes severe economic loss.” “In my opinion, there is no compensation for the damaged crops.”	Economic loss
Conflict among stakeholders (Human–human conflict)	Mistrust	“There are times, we cannot rely on others for conservation… I just cannot trust them” “Have you asked [names]? Did they mention my name?	Emotions: angry, fear, and sad
	Inefficient communication	“I have stayed here more than 40 years, but I think conservation agencies hardly listen to the villagers’ opinions” “I think we need consistent conservation activities… the volunteers also need to be acknowledged for participating in conservation programs… but I am not sure to whom we should tell this.”	Absent of interactive platform
Conservation issues	Limited finance	“We are lacking budget to get appropriate conservation tools in monitoring the wildlife” “Other countries have advance tools to monitor their animals, but we are still facing difficulties to even quantify the animal population here”	Finance for acquiring appropriate monitoring tools.
	Inadequate human resource	“There are limited number of staffs working to monitor wildlife and enforce conservation rules”	HWW assist conservation works.
	Competing interests	“I know it is important to protect the animals here, but the villagers also need improvement in basic infrastructure.” “Economic development should not be carried out considering its negative impacts on the animals.”	Conservation versus socio-economic development
	Ecological barriers	“Major conservation problems are fragmentation and habitat loss due to deforestation.” “Effective method is to reconnect the wildlife corridor (10 lots LKWS), but this is very difficult… There are oil palm estates, private lands, and villagers’ houses.” “There are villagers who own small pieces of land. How much can they contribute to wildlife corridors? They only have enough land to build their houses.”	Fragmented animal corridor Concerns of the villagers
	Wildlife crimes	“Despite strict penalty, poaching and killing animals still occur at several lots of LKWS.”	Snaring, encroachment
Stakeholders’ perceptions of animals	Animals increase (62%)	“Conservation agencies are conducting extensive programs to protect the animals, so the animal should increase.” “Nowadays, the government has enforced additional (severe) penalty for wildlife crimes, people cannot simply hunt. So, the animals should increase in number.”	Local perceptions contradict biological survey
	Animals decrease (38%)	“Based on my observation working in tourism, it is harder to see the animals here… I think the animal has reduced”	Local perception
Stakeholders’ recommendations	Reconnect wildlife corridor	“The long term solution is to connect the fragmented 10 lots of LKWS… But we need everyone’s support to do this” “Some private owners of oil palm estates are willing to give part of their land for animal corridors.”	Support from community and private sectors
	Encourage villagers’ participation in tourism	“The animals are important for tourism development here. But if the villagers do not benefit from tourism, it may cause reduced support for conservation.”	Tourism as incentive for conservation
	Social values	“We need to protect the animals so that the young generation able to see orangutan and proboscis monkey in natural habitat.” “The villagers need to feel that they are appreciated for taking part in conservation programs.” “The motivation is not all about money. Acknowledgement before and after participating in conservation activities, such as giving them certificates.”	Altruistic value, moral obligation, motivation, recognition.
	Culture and norm	“It is our tradition to hunt animals for livelihoods, catch fish, plant hill rice, and collect forest products… But conservation rules restrict our traditional activities.”	Traditional activities
	Conservation programs	“The animal population and habitat availability are not shared during awareness campaigns… these information should be made available to the villagers.”	Conservation message

* Notes are written during interviews and participant observation.

**Table 3:** Main approaches, barriers, and recommendations to improve wildlife conservation.

Approaches	Objectives	Limitations	Integration of Social Elements	Proposed Solutions Based on Stakeholder Perspectives
Awareness programs	To promote public awareness and understanding of wildlife and habitat conservation.	Focus on increased knowledge rather than behavioral change.	Improve individual moral values, beliefs, and attitudes using a non-monetary type of appreciation. To promote personal compassion and tolerance towards animals.	To include scientific information of wildlife and habitat studies during conservation awareness programs. Monitor impact of educational programs on behavioral changes.
Electrical fences	To prevent human–wildlife conflict: loss of agricultural yield, housing damage, and killing of animals.	Difficult to install fences, and they carry high maintenance cost. Disrupt wildlife movements and creates bottlenecks, worsening conflicts in some situations.	Individual knowledge, obligations, and ethics. To encourage individual responsibility to maintain already built fences.	To build integrated electrical fences between different blocks of private land.
Tree-planting	Plant trees along Lower Kinabatangan River in most degraded forest areas.	Require support from the local community to carry out the project. Limited finance, tools, and human resources are a major conservation issue.	Individual moral values and attitudes To encourage local participation in habitat restoration.	The project allows villagers to earn income by selling plant seedlings to Rileaf. This initiative should be extended to include non-participant villagers.
Honorary Wildlife Warden (HWW)	Local community members are elected and trained by the Sabah Wildlife Department and have the legal powers to apprehend offenders.	Most appointments are individuals already working in conservation or tourism. They are not remunerated by the state. Limited numbers of HWW staff.	Individual value, knowledge, perception, and attitudes. To increase local support for conservation through a non-monetary approach.	To increase individual participation in volunteer reporting of wildlife crimes, improve compassion through certificates of acknowledgement, and reduce dependency on HWW.
Wildlife corridor	To reconnect fragmented animal movement routes and restore habitat.	Challenges to connect fragmented areas due to various land use activities.	Individual moral values, knowledge, and attitudes. To encourage local participation in habitat restoration, including the private sector (oil palm plantations).	To encourage strong support and participation from oil palm plantation owners in constructing wildlife corridor.

## Data Availability

The data presented in this study are available on request from the corresponding author. The data are not publicly available due to containing information that could compromise research participant consent.

## Supplementary Materials

The following supporting information can be downloaded at: https://www.mdpi.com/article/10.3390/ani12070811/s1. File S1: Interview questions.
