# Supplementary material for: Understanding Social Dimensions in Wildlife Conservation: Multiple Stakeholder Views
Source: Animals (Basel). 2022 Mar 23;12(7):811. doi: 10.3390/ani12070811 (PMC8996913; doi:10.3390/ani12070811)
Supplement: Supplementary file 1 [file animals-12-00811-s001.zip › animals-1573659-supplementary.pdf]

animals

MDPI

Supplementary Material

Understanding Social Dimensions in Wildlife Conservation: Multiple Stakeholder Views

Marcela Pimid [1], Mohammad Rusdi Mohd Nasir [1,*], Kumara Thevan Krishnan [2,*], Geoffrey K. Chambers [3], A Ghafar Ahmad [4] and Jimli Perijin [5]

1 Faculty of Architecture and Ekistics, Universiti Malaysia Kelantan, Bachok 16300, Malaysia; marcela.fun@gmail.com
2 Faculty of Agro Based Industry, Universiti Malaysia Kelantan, Jeli 17600, Malaysia
3 School of Biological Sciences, Victoria University of Wellington, P.O. Box 600, Wellington 6140, New Zealand; geoff.chambers@vuw.ac.nz
4 School of Housing, Building, and Planning, Universiti Sains Malaysia, Minden 11800, Malaysia; aghafar7788@yahoo.com
5 Sabah Wildlife Department, Kota Kinabalu 88100, Malaysia; jimliowon@gmail.com
* Correspondence: rusdi.mn@umk.edu.my (M.R.M.N.); thevan@umk.edu.my (K.T.K.)

Citation: Pimid, M.; Nasir, M.R.M.; Krishnan, K.T.; Chambers, G.K.; Ahmad, A.G.; Perijin, J. Understanding Social Dimensions in Wildlife Conservation: Multiple Stakeholder Views. *Animals* **2022**, *12*, 811. https://doi.org/10.3390/ani12070811

Academic Editors: Daniel Ramp

Received: 12 January 2022
Accepted: 17 March 2022
Published: 23 March 2022

Publisher's Note: MDPI stays neutral with regard to jurisdictional claims in published maps and institutional affiliations.

Copyright: © 2022 by the authors. Licensee MDPI, Basel, Switzerland. This article is an open access article distributed under the terms and conditions of the Creative Commons Attribution (CC BY) license (https://creativecommons.org/licenses/by/4.0/).

Interview Questions

Section S1: Situation Assessment

1. What do you think about the local conservation awareness?
2. What do you think about conservation and tourism in Kinabatangan?
3. What factors influence local support for conservation?
4. How is the progress of Lower Kinabatangan Wildlife Sanctuary?
5. What do you think about the number of wildlife in Kinabatangan?
6. What do you think about the size of habitat available for the animals?
7. What do you think about the human-wildlife conflict?

Section S2: Decision-Making

1. How does the decision-making conducted for animal conservation?
2. What do you think about the relationship among conservation management in protecting the animals?
3. What do you think about the relationship between the community and conservation management?

Section S3: Monitoring and Evaluation

1. What factors influence conservation outcomes?
2. Are you willing to support conservation programs in the future?
3. What is your opinion about human values impact on animal conservation?
4. What do you suggest to improve current conservation approaches?
